# Chemically Functionalized Single-Walled Carbon Nanotubes Prevent the Reduction in Plasmalemmal Glutamate Transporter EAAT1 Expression in, and Increase the Release of Selected Cytokines from, Stretch-Injured Astrocytes in Vitro

**DOI:** 10.3390/cells13030225

**Published:** 2024-01-25

**Authors:** Nika Gržeta Krpan, Anja Harej Hrkać, Tamara Janković, Petra Dolenec, Elena Bekyarova, Vladimir Parpura, Kristina Pilipović

**Affiliations:** 1 Department of Basic and Clinical Pharmacology and Toxicology, Faculty of Medicine, University of Rijeka, HR-51000 Rijeka, Croatia; nika.grzeta@uniri.hr (N.G.K.); aharej@uniri.hr (A.H.H.); tamara.jankovic@medri.uniri.hr (T.J.); petra.dolenec@medri.uniri.hr (P.D.); 2 Department of Chemistry, University of California, Riverside, CA 92521, USA; elenab@ucr.edu; 3 International Translational Neuroscience Research Institute, Zhejiang Chinese Medical University, Hangzhou 310053, China; vlad@zcmu.edu.cn

**Keywords:** astrocytes, brain injury, traumatic, cytokines mouse, nanotubes, carbon

## Abstract

We tested the effects of water-soluble single-walled carbon nanotubes, chemically functionalized with polyethylene glycol (SWCNT-PEG), on primary mouse astrocytes exposed to a severe in vitro simulated traumatic brain injury (TBI). The application of SWCNT-PEG in the culture media of injured astrocytes did not affect cell damage levels, when compared to those obtained from injured, functionalization agent (PEG)-treated cells. Furthermore, SWCNT-PEG did not change the levels of oxidatively damaged proteins in astrocytes. However, this nanomaterial prevented the reduction in plasmalemmal glutamate transporter EAAT1 expression caused by the injury, rendering the level of EAAT1 on par with that of control, uninjured PEG-treated astrocytes; in parallel, there was no significant change in the levels of GFAP. Additionally, SWCNT-PEG increased the release of selected cytokines that are generally considered to be involved in recovery processes following injuries. As a loss of EAATs has been implicated as a culprit in the suffering of human patients from TBI, the application of SWCNT-PEG could have valuable effects at the injury site, by preventing the loss of astrocytic EAAT1 and consequently allowing for a much-needed uptake of glutamate from the extracellular space, the accumulation of which leads to unwanted excitotoxicity. Additional potential therapeutic benefits could be reaped from the fact that SWCNT-PEG stimulated the release of selected cytokines from injured astrocytes, which would promote recovery after injury and thus counteract the excess of proinflammatory cytokines present in TBI.

## 1. Introduction

Traumatic brain injury (TBI) represents a significant public health problem due to the fact that it is one of the leading causes of death and disability worldwide [1]. Pathological changes that occur in the brain following trauma result from the primary, irreversible injury and the subsequent secondary injury mechanisms that develop over time. According to the Glasgow Coma Scale (GCS), a classification based on the neurological status of patients, there are three degrees of severity of TBI, mild, moderate, and severe. Severe TBI has a 30–40% mortality rate and it can cause significant physical, psychosocial, and social deficits in the majority of surviving patients [2]. Survivors, after moderate and severe TBI, have significant long-term deficits that develop due to the widespread cellular damage and the neurotoxic environment around the site of the injury, the latter in part due to excessive glutamate [3], as well as prolonged and intense neuroinflammation mediated by cytokines [4]. There are no effective treatments to help mediate the repair and regeneration of the brain tissue after TBI. The current therapeutic approach for TBI solely relies on acute stabilization of the patient and administration of supportive therapy [5].

It is known that TBI, as a part of the tissue response to injury, induces the activation of astrocytes, one of the most abundant glial cells in the brain. This process, known as reactive astrogliosis, is involved in tissue remodeling, repair of the blood–brain barrier (BBB), regulation of synaptic plasticity, formation of a glial scar, and changes in the extracellular matrix [6]. Reactive astrocytes are characterized by molecular, cellular, and functional changes that arise in the response to brain damage. A common feature of this type of reaction is cellular hypertrophy and upregulation of the glial fibrillary acidic protein (GFAP) expression [7]. Indeed, GFAP is one of the key markers for the activation of reactive astrogliosis following brain injury or other types of stress in the central nervous system (CNS).

Astrocytes are the major buffering cells for the excitatory amino acids (EAAs), in particular glutamate, that is abundantly released from the injured neuronal cells [8]. Removal of glutamate from the extracellular space via astrocytic plasmalemmal glutamate transporters, which belong to the excitatory amino acid transporter (EAAT) family, is crucial for glutamate homeostasis. Glutamate uptake by astrocytes prevents elevation of glutamate to excitation levels in the extracellular space, which is a critical factor for the survival of neurons [9,10]. Two major types of Na^+^-dependent glutamate transporters in astrocytes of the CNS are EAAT1 (in rodents also referred to as GLAST) and EAAT2 (in rodents also referred to as GLT-1) [11]. Their expression is developmentally regulated, so in neonatal mice, which we used here, astrocytes express low levels of GLT-1/EAAT 2 when compared to GLAST/EAAT1 expression [12]. In both preclinical and clinical studies, significant dysregulation of these transporters following TBI was detected [13,14,15]. Of particular interest to our present study is the fact that there is a loss of EAAT1 and 2 expression in astrocytes in human patients suffering from TBI [15]. Taming this loss may provide a fertile ground for therapeutic intervention.

Carbon nanotubes (CNTs) may be employed in this quest. CNTs are promising nanomaterial candidates in treating brain disorders [16]. They have excellent physicochemical properties and the ability to interact with neurons, neuronal circuits, and astrocytes. Most of the previous studies have been focused on bio-imaging and drug delivery applications of CNTs, whereas their application as a therapeutic agent has not yet been sufficiently investigated [16]. Some difficulties regarding the widespread applications of CNTs in treating disorders of the CNS are related to their biocompatibility. Namely, especially with pristine, non-functionalized CNTs, it was detected that they can increase free radical production, the accumulation of which leads to oxidative stress; induce tissue inflammatory responses; and cause DNA damage [16]. However, these issues can be alleviated by changing the physicochemical properties of the CNTs, making them more prone to dispersion in the aqueous media and thus enhancing their biocompatibility.

SWCNTs chemically functionalized with polyethylene glycol (PEG) or poly-m-aminobenzene sulfonic acid are aqueous colloidal solutes. These nanomaterials, when applied on a short term in cell culture (3 days), promoted outgrowth of selected neurites [17] and induced changes in the morphology of astrocytes by enhancing the GFAP expression, as measured by immunostaining, and caused the appearance of a more mature phenotype of astrocytes [18]. Furthermore, SWCNT-PEG (in cell culture for 4 days), but not PEG itself, caused an increase in expression of GLAST/EAAT1 in astrocytes along with an increase in the astrocyte uptake of this excitatory transmitter [19].

It is still largely unknown how the traumatically injured astrocytes would react to exposure to water-soluble chemically functionalized SWCNTs. Until now, only one study reported the effects of the application of SWCNT-PEG (for a longer term of 28 days) in a model of the traumatic injury of the CNS in vivo [20]. The study showed that the injection of SWCNT-PEG at the injury site in rats with complete transection of the spinal cord, at the thoracic nine level, decreased the lesion volume and modestly improved recovery of hind limb locomotor function, and without the change in GFAP expression.

The aim of this study was to initially investigate the effects of SWCNT-PEG on the survival of astrocytes exposed to severe in vitro (cell culture) TBI within the first 24 h and then to subsequently study if SWCNT-PEG caused changes in the protein expression of GFAP and EAAT1 in the injured astrocytes. Furthermore, we also examined if there were significant changes in the secretome profile of these cells, which was analyzed by using the multiplex cytokine array, in the media collected from cultures of injured astrocytes. It should be noted that the stretch injury as a model of TBI used here does not produce the overt necrosis that may be present in whole-animal TBI. Nonetheless, our study provides for the profile with respect to the use of SWCNT-PEG as a potential curative agent acting upon injured astrocytes.

## 2. Materials and Methods

### 2.1. Experimental Animals

Mice used for this research were born and bred in the Laboratory for Mice Breeding and Engineering Rijeka (LAMRI). For the preparation of the in vitro culture of astrocytes, C57BL/6 mice of both sexes at 0–2 days of age were sacrificed. For each analysis, three to four groups of mice from different mothers were sacrificed on different days for the isolation of astrocytes to perform experiments in biological triplicates or quadruplicates. All the procedures on mice were performed at the Department of Basic and Clinical Pharmacology and Toxicology of the Faculty of Medicine of the University of Rijeka. The use of animals for experiments was approved by the Faculty’s Animal Welfare Committee, as well as by the Veterinary Department of the Ministry of Agriculture, and the experiments were performed in accordance with the Croatian Animal Protection Act, which was matched with the current European Union legislation.

### 2.2. In Vitro Experiments

#### 2.2.1. Astrocyte Culture

Astrocytes were isolated from mice neocortices with some modifications of a previously described procedure [21]. After removing mice brains, neocortices were isolated, cleaned of meninges, and put into ice-cold cell culture medium lacking phenol-red (Dulbecco’s Modified Eagle Medium, DMEM; Cat. No. P04-01161, PAN-Biotech, Aidenbach, Germany). Neocortices were homogenized with an automatic pipettor and then passed through 230 μm and 140 μm pore-diameter sterile filters, respectively. The filtrate was pelleted by centrifugation, the supernatant was decanted, and the pellet was resuspended in 2.5 mL of completed medium containing DMEM with phenol-red (Cat. No. P04-04510) and 10% (*v*/*v*) fetal bovine serum (FBS) (both from PAN-Biotech, Germany) with added 1% *v*/*v* of antibiotic–antimycotic solution (Cat. No. A5955; Sigma-Aldrich, Saint Louis, MO, USA). The resulting cell suspension was passed through a 73 μm pore-diameter sterile filter. Cells in the filtrate were counted and seeded at a density of 2 × 10^5^ cells/cm^2^ into 75 cm^2^ flasks and incubated at 37 °C in a 5% CO_2_—95% air atmosphere incubator with relative humidity of 95% for 7 days. At that time, flasks were shaken twice on an orbital incubator–shaker (Unimax 1010 Orbital Platform Shaker, Heidolph, Schwabach, Germany), first for 30 min at 180 rpm, and then, after replacing the media, again for 6 h at 240 rpm. The supernatant was discarded, the fresh medium was added to the adherent cells, i.e., astrocytes, and flasks were then put back into the incubator for an additional 7–14 days until they were used in experiments. This procedure yields purified, ~99% astrocytes culture, as confirmed using immunocytochemistry on GFAP and EAAT1 (Supplementary Materials; Figure S1).

#### 2.2.2. Cell Injury Model

Purified astrocytes were detached from flasks and after mechanical trituration seeded into wells bottomed with silastic membranes that were top-coated with collagen type I (BioFlex^®^ culture plates, Cat. No. BF-3001C; FlexCell^®^ International Corporation, Burlington, NC, USA). We applied 150,000 cells in 2.5 mL growth medium per well. Upon reaching confluence (within three to five days), astrocytes were subjected to injury by the controlled delivery of pressurized nitrogen. Pressure was regulated by the Cell Injury Controller II (Virginia Commonwealth University, Richmond, VA, USA). We applied a 19–29 kPa (3.8–4.2 psi) peak pressure pulse, 50 ms in duration, perpendicular to the silastic membrane. This caused a membrane displacement of 7.5 mm and biaxial stretch of the cells attached to them. This injury in vitro corresponds to the severe traumatic brain injury (sTBI) in vivo [22]. After the injury, the cells were returned to the incubator.

#### 2.2.3. Single-Walled Carbon Nanotubes Preparation

Purified single-walled carbon nanotubes were obtained from Carbon Solutions Inc., Riverside, CA, USA (Cat No. P3-SWNT; www.carbonsolution.com (accessed on 15 January 2024)) and functionalized with PEG, as described in detail in the Supplementary Materials. The attached PEG functionalities rendered the carbon nanotubes soluble in purified water upon ultrasonication for 1 h. The stock SWCNT-PEG aqueous colloidal solution was at 2 mg/mL. We used SWCNT-PEG due to their known biological effects on astrocytes, as we disclose in the introduction [17,18,19,20]. We characterized SWCNT-PEG with atomic force microscopy, thermogravimetric analysis, mid-infrared spectroscopy, and ultraviolet-visible–near-infrared spectroscopy (Supplementary Materials; Scheme S1 and Figures S2–S5) to determine their morphology and chemical composition. The SWCNT-PEG material contains 72.3 weight percent (wt%) SWCNTs, 22.6 wt% PEG, and 5.1 wt% metal nanoparticles (nickel and yttrium mixture, in the atomic ratio of 4:1). SWCNT-PEG exist as individual tubes as well as axially aligned small bundles. The individual SWCNT-PEG have diameters between 1.2 nm and 1.7 nm with an average diameter of 1.5 nm, while bundles have a diameter of 2 nm to 7 nm, and the length in the range of 0.3–1.5 µm.

#### 2.2.4. Incubation of the Primary Astrocyte Culture with SWCNT-PEG or the Functionalization Agent PEG

SWCNT-PEG were added to the astrocyte cultures 1 h after the injury at a concentration of 5 μg/mL and kept for 23 h. This concentration was chosen according to the previous research using this very material, for 3 or 4 days on cultured astrocytes [18,19], showing its biocompatibility along with the effect on astrocytes, which included increases in GFAP and EAAT1 levels, respectively, as measured by immunostaining, and functionally enhanced uptake of glutamate by these glial cells [19]. Before adding SWCNT-PEG to astrocytes with sTBI (sTBI+CNT group), the stock solution was diluted in 150 μL of the cell culture medium and applied to the culture medium to reach the final concentration of 5 μg/mL. In uninjured astrocytes (control group) and sham-treated astrocytes with sTBI (sTBI group), instead of SWCNT-PEG stock solution, we used purified water containing the functionalization agent PEG, to mix in with 150 μL of the cell culture medium to reach the final concentration of 1 μg/mL of PEG, as previously described [23]. This concentration of PEG corresponds to the PEG weight load in SWCNT-PEG. The final ratio of purified water (in PEG or SWCNT-PEG solution) to cell culture medium was 1:400. Thus, we had three experimental groups: (i) control, uninjured astrocytes receiving PEG; severe TBI (sTBI), injured astrocytes receiving PEG; and sTBI+CNT, injured astrocytes receiving SWCNT-PEG.

### 2.3. Determination of Severity of the Astrocyte Injury

For the determination of the severity of the astrocyte injury, release of lactate dehydrogenase (LDH) into cell culture media was measured using the CytoTox96^®^ Non-Radioactive Cytotoxicity assay (Promega Corporation, Madison, WI, USA). Aliquots of astrocyte culture media were collected at 15 min, 1 h, 3 h, 6 h, and 24 h after the injury, and transferred into a 96-well plate for sample analysis. Samples were treated with the reagents from the kit in accordance with the manufacturer’s instruction manual and analyzed by the spectrophotometric method using the BioTek EL808 Microplate Reader (Agilent Technologies, Santa Clara, CA, USA).

### 2.4. Dot Blot

The dot blot method was used to detect the levels of oxidatively damaged proteins by measuring the protein carbonyl content. 2,4-dinitrophenylhydrazine (DNPH) reacts with the carbonyl group on the protein to form 2,4-dinitrophenylhydrazone (DNP hydrazone), which is detected immunochemically. Two aliquots of each sample were used, one of which was subjected to the derivatization reaction, while the other aliquot represented the negative control. Protein samples were derivatized with 10 mM DNPH in the presence of 12% sodium dodecyl sulfate (SDS) for 15 min at room temperature (20–25 °C) in the dark. A neutralization solution (2 M Tris, 30% glycerol, and 19% 2-mercaptoethanol) was then added to the samples, which were then spotted onto a polyvinylidene difluoride (PVDF) membrane using a dot blot device (Cleaver Scientific Ltd., Warwickshire, UK). The membrane was dried for 15 min and washed twice in succession with concentrated acetic acid and then water. The membrane was then rinsed with wash buffer (10 mM Tris-HCl, pH 7.5, 150 mM NaCl, 0.05% Tween; i.e., TBST), blocked by incubation with 5% bovine serum albumin (BSA) in TBST for one hour. After blocking, an anti-DNP antibody (OxyBlot™, EMD Millipore, Billerica, MA, USA), diluted 1:100 in TBST with 1% BSA, was added to the membranes and kept overnight at 4 °C. The next day, the membrane was washed 3 times for 5 min with TBST and then was incubated with biotinylated goat anti-rabbit secondary antibody (1:3000) (Table 1) for one hour at room temperature. After that, the membrane was washed with TBST again and incubated with streptavidin conjugated with horseradish peroxidase (HRP). A detection solution (chemiluminescent substrate SuperSignal™ West Pico PLUS; Thermo Fisher Scientific, Waltham, MA, USA) was added to the membrane and visualized on a Kodak Image Station 440CF device, analyzed in Kodak 1D Image Analysis Software, Version 3.6 (Eastman Kodak, Rochester, NY, USA).

### 2.5. Western Blotting

For the Western blotting, samples were prepared by lysing the cells at 24 h after the injury with 100 μL/well of ice-cold radioimmunoprecipitation assay (RIPA) buffer, complemented with protease and phosphatase inhibitors (cOmplete^TM^, Cat. No. 11873580001, Roche, Basel, Switzerland). Cells were scraped from the silastic membranes and placed in a vessel on the orbital shaker on ice for 20 min. The resulting cell lysate was collected, and cell debris pelleted by centrifugation at 4 °C. The supernatants were collected and stored at −80 °C. The concentration of proteins in supernatants was determined using the Bradford method [24].

Proteins were loaded onto 10% polyacrylamide gels at a concentration of ~1 μg/μL. The electrophoresis was run at 200 V in the Mini-PROTEAN^®^ Tetra System (Bio-Rad Laboratories Inc., Hercules, CA, USA). The transfer of proteins from gels to nitrocellulose membranes was performed in the Trans-Blot^®^ Turbo™ Transfer System (Bio-Rad Laboratories Inc., Hercules, CA, USA). Afterwards, membranes were treated for 1 h with 5% dry milk or 5% BSA in TBST to block non-specific binding sites. The primary antibodies used were mouse anti-GFAP, rabbit anti-EAAT1, and mouse anti-β-actin (all at 1:1000) (Table 1); β-actin stain was used as a loading control. After the overnight incubation at 4 °C, membranes were washed with Tris-buffered saline (TBS) containing 0.1% Tween (TBST2) for 30 min and incubated with secondary antibodies for 1 h. The secondary antibodies used were goat anti-rabbit IgG and goat anti-mouse IgG, both conjugated with biotin (Table 1). After the incubation, membranes were again washed with TBST2 and then incubated with the streptavidin–HRP conjugate for 30 min. Visualization of proteins, achieved by adding the chemiluminescent substrate SuperSignal™ West Pico PLUS (Thermo Fisher Scientific, Waltham, MA, USA), was performed using the Kodak Image Station 440; bands were analyzed using the Kodak 1D program (v.3.6.5, Kodak Scientific Imaging Systems, Kennewick, WA, USA).

### 2.6. Cytokine Assay

For the measurement of cytokines released by astrocytes, cell culture media aliquots were collected at 24 h after the injury. Media samples were screened for 62 or 40 cytokines by using the Mouse Cytokine Array C3 kit (for data in Figure 4 and Table S1) or Mouse Inflammation Array C1 kit (for data in Figure S7 and Table S2), respectively, and according to the manufacturer’s instructions (Ray Biotech Inc., Norcross, GA, USA). In brief, membranes provided in the kit were sequentially incubated with equal volumes (200 µL) of the cell culture medium, primary biotinylated antibodies and HRP-conjugated streptavidin solution. Visualization of signals on membranes was performed on a Kodak Image Station 440 and their relative intensities were analyzed using the Kodak 1D program (v. 3.6.5, Kodak Scientific Imaging Systems, USA).

### 2.7. Statistical Analyses

All analyses were performed on at least three separate, independent experiments (biological replicates). The sample size required for an individual set of experiments was pre-assessed using power analysis (set at 80% and α = 0.05), and the normality of data was established using the Shapiro–Wilk test for normality. For all the statistical analyses, Statistica software version 13.0 (StatSoft, Inc., Tulsa, OK, USA) was used. Comparisons between multiple groups were done using one-way analysis of variance (ANOVA) followed by the Duncan’s post hoc multiple comparison test. Results are expressed as mean ± standard error of means (SEM). In all the comparisons, *p* < 0.05 was considered to indicate statistical significance. To remove the variations between the Western blotting sessions, the results of the densitometric analyses were corrected with the Factor Correction program version 16.1 [25].

## 3. Results

In our experiments, astrocytes were plated on silastic membranes and, upon reaching confluence, exposed to stretch injury simulating a severe TBI. Astrocytes were then treated with 5 μg/mL SWCNT-PEG (sTBI+CNTs group), or 1 μg/mL PEG (sTBI group), starting from 1 h post-injury for 23 h; see Materials and Methods for concertation considerations for SWCNT-PEG and PEG. Control astrocytes that were uninjured received 1 μg/mL PEG (control group) according to the above schedule. The addition of PEG to both sTBI and control groups provided assurance that the effects we observed in the sTBI+CNT group were the result of the SWCNT backbone action, rather than that of PEG, as a functionalization agent. We assessed the effects of SWCNT-PEG on the astrocyte viability and cell protein oxidative damage upon injury. We also investigated if exposure to SWCNT-PEG changes the expression of proteins GFAP and EAAT1, and if this nanomaterial affects the release of cytokines from the injured astrocytes.

### 3.1. SWCNT-PEG Do Not Affect Cell Viability of Astrocytes Exposed to Severe Stretch Injury

The therapeutic potential of CNTs is somewhat hampered by their possible toxicity, although it has been shown that their harmfulness is considerably lower in cases of purified and surface-functionalized nanotubes [26]. Specifically, SWCNT-PEG, the very material we use here, did not exert adverse effects on astrocytes when used at a concentration of 5 μg/mL for up to 4 days in culture [19]. The effects of SWCNT-PEG on the viability of astrocytes exposed to severe in vitro trauma have not been assessed until now.

**Figure 1:**
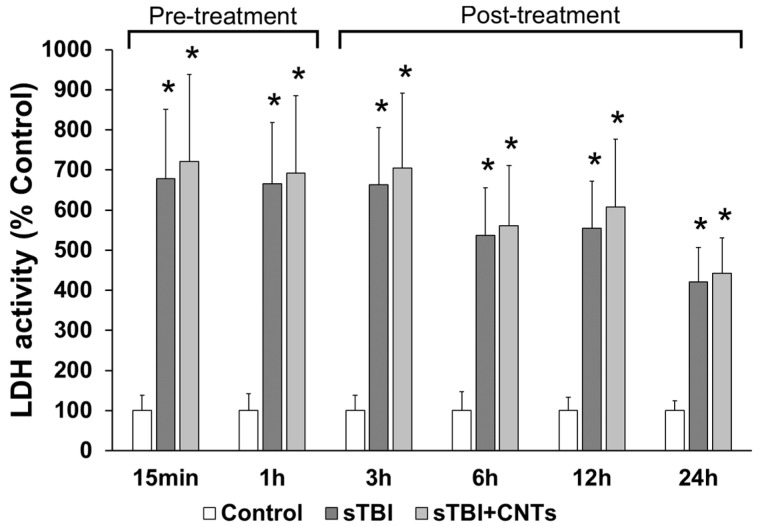
The lack of effect of chemically functionalized single-walled carbon nanotubes (CNTs) on the survival of the mouse primary astrocytes exposed to severe traumatic brain injury (sTBI) in vitro. Injured cells were treated with PEG (sTBI) or SWCNT-PEG (sTBI+CNTs) at 1 h post-stretch injury. Cell culture media were collected at different time points post-injury (15 min, 1 h, 3 h, 6 h, 12 h, and 24 h), from which lactate dehydrogenase (LDH) activity was quantified. Results are expressed as means ± SEM (N = 3). * *p* < 0.05, significantly different from the control (uninjured and PEG-treated) group (one-way ANOVA). There was not a significant change in LDH activity over time in the control group.

We assessed the cytotoxicity by measuring the plasma membrane permeability to LDH by quantifying LDH activity in the cell culture medium, as this cytosolic enzyme diffuses (through a leaky membrane) into the extracellular space. Control, uninjured astrocytes show low levels of LDH at all time points recorded, prior to and after they received the PEG treatment (Figure 1). This is in a stark contrast to astrocytes that received a severe stretch injury. In order to establish that the level of cell injury between the experimental conditions was comparable, LDH levels were first measured at 15 min and 1 h after the in vitro TBI, i.e., before the application of either SWCNT-PEG or PEG at 1 h post in vitro TBI. LDH concentrations were additionally determined at 3 h, 6 h, 12 h, and 24 h post-injury and with SWCNT-PEG or PEG added to cells. At all the investigated time points, LDH levels were significantly higher in the culture media of the injured cells compared to the control conditions (*p* < 0.05). Exposure to either SWCNT-PEG or PEG did not affect the amount of LDH released from the injured astrocytes at any of the designated time points (*p* > 0.05).

### 3.2. Severe Stretch Injury of Cultured Astrocytes and the Application of SWCNT-PEG Do Not Cause Changes in the Protein Oxidation Levels

**Figure 2:**
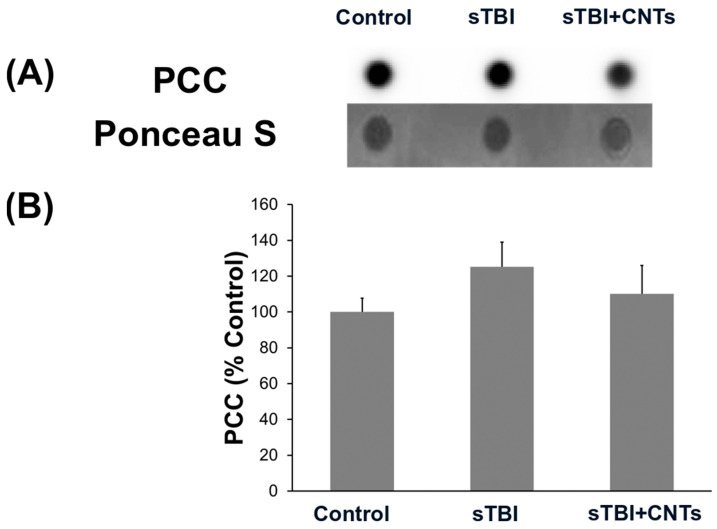
The lack of effect by chemically functionalized single-walled carbon nanotubes (CNTs) on the levels of oxidative protein damage, determined by the measurement of the protein carbonyl content (PCC), in mouse primary astrocytes exposed to severe traumatic brain injury (sTBI) in vitro. Shown are (**A**) representative dot blots and (**B**) the results of the densitometric analyses. The histogram shows the PCC levels in control (uninjured cells receiving PEG) conditions, as well as at 24 h after the injury and following the addition of PEG (sTBI) or SWCNT-PEG (sTBI+CNTs). Values are shown as the proportion (%) of PCC levels detected in the cells of the control group, corrected for the protein load determined by Ponceau S staining. Results are expressed as means ± SEM. Results of the analyses from four separate biological replicates are presented.

Protein carbonyl levels were detected using the dot blot immunoassay to monitor if the oxidative damage of proteins is potentially caused by the in vitro severe TBI and/or the SWCNT-PEG treatment (Figure 2). Figure 2A shows representative dot blots of experimental group samples, and Figure 2B the associated densitometric analyses corrected for the protein load determined by Ponceau S staining and presented as a percentage of the results from the control group samples. It is evident from the results that there is no statistically significant change in the levels of carbonylated proteins following cell injury or the addition of the investigated nanomaterial (or functionalization agent), determined at 24 h after severe cell stretch injury.

### 3.3. Application of SWCNT-PEG to Astrocytes Rescues the Injury-Induced Loss of Plasmalemmal Glutamate Transporter EAAT1

**Figure 3:**
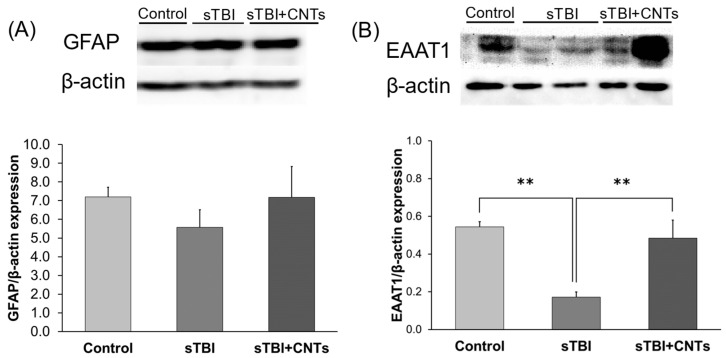
The effects of chemically functionalized single-walled carbon nanotubes (CNTs) on the protein expression of GFAP and EAAT1 in mouse primary astrocytes exposed to severe traumatic brain injury (sTBI) in vitro. Injured cells were treated with PEG (sTBI) or SWCNT-PEG (sTBI+CNTs) at 1 h post-stretch injury. Cells were lysed at 23 h after the treatment. Densitometric analyses of (**A**) GFAP and (**B**) EAAT1 expressions. There was no change in the expression of GFAP protein in injured cells, both treated with SWCNT-PEG or PEG, in comparison with the control cells. Injury significantly decreased levels of EAAT1 in cells treated with PEG in comparison to control cells, while the addition of SWCNT-PEG restored injury-induced loss of EAAT1 to a level similar to that seen in the uninjured, PEG-treated control. Results of the densitometric analyses were corrected for the β-actin contents. Data are expressed as mean ± SEM. ** *p* < 0.001 (one-way ANOVA).

Changes in the expression of GFAP following brain injury are the most characteristic markers of astrocyte reactivity [7]. In our investigation, Western blot analyses of GFAP protein expression (Figure 3A) showed no significant change in GFAP levels at 24 h after severe stretch injury (F(2;8) = 0.583; *p* = 0.580). This is in line with the previously published research by Wanner et al. [27] in which no significant increase in GFAP synthesis, but mainly induction of relocalization of existing GFAP in astrocytes, was detected. In our experiment, the addition of nanotube colloid solution also did not affect the GFAP content, which is in agreement with the previously published work in spinal cord in vivo injury treated with SWCNT-PEG for 28 days [20], but at odds with an increase in the immunoreactivity of GFAP in cultured astrocytes exposed to SWCNT-PEG for 3 days [19].

In human patients suffering from TBI, there is a loss of EAAT1 expression in astrocytes [15]. We first confirmed that our stretch-injury model appropriately simulates such EAAT1 expression in vitro. We then assessed whether we can revert this loss by using SWCNT-PEG as a proof-of-principle demonstration of the translational use of this nanomaterial. Namely, it has been previously established that SWCNT-PEG cause an increase in the immunoreactivity of glutamate transporter EAAT1 on the plasma membrane of astrocytes [19].

We, therefore, evaluated if the application of the investigated nanomaterial to astrocytes exposed to severe stretch impacts the total expression level of EAAT1, and the results of this analysis are presented in Figure 3B. Analyses showed that the expression of EAAT1 was affected by severe stretch injury (F(2;9) = 11.157; *p* = 0.004). Post hoc comparison revealed that the in vitro trauma causes a decrease in the EAAT1 levels (*p* = 0.002) and that the addition of the SWCNT-PEG, but not PEG, significantly rescues the injury-induced loss of this plasmalemmal glutamate transporter (*p* = 0.005). This finding points to a possible translational use of SWCNT-PEG to treat injured astrocytes in the attempt to rescue their ability to uptake the excess of extracellular glutamate that occurs in TBI.

We separately conducted experiments in which we tested whether SWCNT-PEG treatment affects GFAP or EAAT1 expressions in astrocytes not exposed to the stretch paradigm, but no significant differences were detected in levels of both investigated proteins. between control, non-treated, and PEG- or SWCNT-PEG-treated astrocytes (Figure S6).

### 3.4. Cytokine Array

Astrocytes are considered secretory cells of the CNS, and their ability to secrete cytokines can be associated with a variety of inflammatory roles and pathologies [28,29]. Thus, we assessed the secretion of cytokines from astrocytes in our three experimental groups.

**Figure 4:**
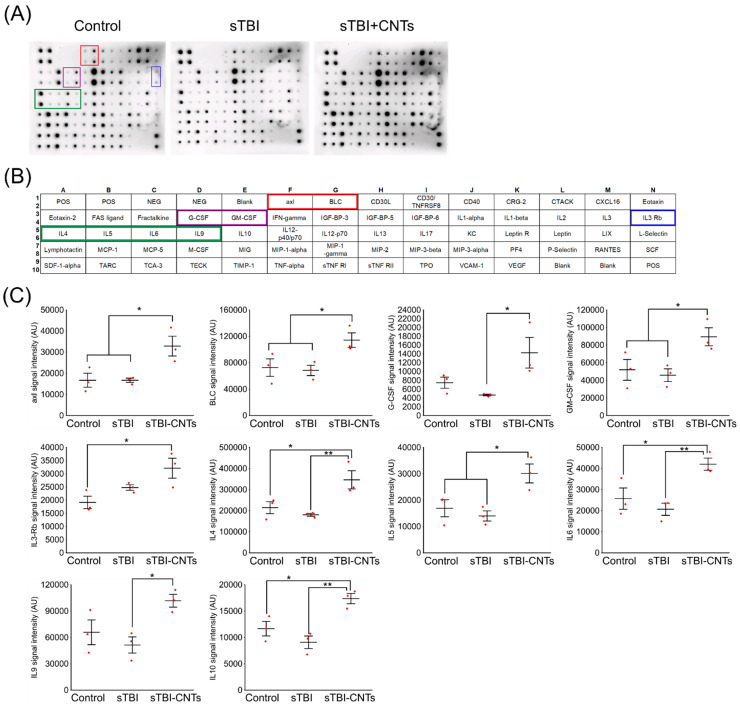
Chemically functionalized single-walled carbon nanotubes (CNTs) increase the secretion of selected cytokines from astrocytes exposed to severe traumatic brain injury (sTBI) in vitro. (**A**) Representative membranes showing the content/pattern of cytokines, released from the control group of astrocytes, i.e., uninjured and PEG-treated cells (control), astrocytes subjected to stretch injury and treated with PEG (sTBI) or treated with SWCNT-PEG (sTBI+CNTs). (**B**) Table with the list of cytokines available within the Mouse Cytokine Array C3 kit. Letters (A–N) and numbers (1–10) indicate coordinates (x,y) on the dot blot. Colored boxes indicate selected proteins with significant changes in their expression. (**C**) Summary graphs showing significant increases in signal intensity of selected (boxed in (**A**,**B**)) cytokines in the cell culture media samples from the SWCNT-PEG-treated injured astrocytes. Data are expressed as mean ± SEM. * *p* < 0.05, ** *p* < 0.001.

The antibody array we used allowed us to assay simultaneously 62 mouse cytokines. Analyses of the media, conditioned by the control (uninjured, PEG-treated) astrocytes, sampled at 24 h after the onset of the experiment, detected the secretion of multiple compounds (Figure 4 and Supplementary Materials, Table S1; the latter table contains all the abbreviations used in the text below). In the media derived from the injured cells treated with PEG, no significant changes in the levels of the cytokines were found compared to the media collected from the control astrocytes. Conversely, results of the microarray analysis of the media conditioned by astrocytes that were subjected to the in vitro severe TBI and subsequently exposed to SWCNT-PEG revealed that the investigated treatment significantly affected the levels of the following compounds: axl, BLC, G-CSF, GM-CSF, IL-3-Rb, IL-4, IL-5, IL-6, IL-9, and IL-10. In the media sampled from the injured cells treated with nanotubes, levels of axl, BLC, GM-CSF, IL-4, IL-5, IL-6, and IL-10 were higher compared to both the concentrations determined in the media conditioned by the control cells and the injured, PEG-treated, astrocytes. Additionally, statistical analyses revealed that the G-CSF and IL-9 levels were increased in the samples from the CNT-treated, injured cells (sTBI+CNTs) only when compared to the results obtained from the PEG-treated, stretched astrocytes. Regarding the IL-3-Rb levels, they were significantly higher in the media sampled from the SWCN-PEG-treated, injured only compared to the media taken from the cells maintained in the control conditions.

In separate experiments, we also tested if the SWCNT-PEG exposure has an effect on astrocytes not exposed to the stretch injury. Other than the decrease in the release of the single cytokine, MIP-1 gamma, from SWCNT-PEG-treated astrocytes compared to control, non-treated cells, no other differences in the cytokine levels were detected between the experimental groups (Figure S7; Table S2). It should be noted that the antibody array kit (see Materials and Methods and compare Tables S1 and S2) used in these experiments did not test the levels of all the affected cytokines in the above TBI experiments (Figure 4 and Table S1), so that axl and IL-5 levels where not tested. However, this technicality should not distract from the fact that SWCNT-PEG stimulated the release of selected cytokines from injured astrocytes, which would promote recovery after injury and thus counteract the excess of proinflammatory cytokines present in TBI.

## 4. Discussion

In the present study, we used an in vitro astrocyte stretch injury model to demonstrate the effects of SWNTC-PEGs on the cell survival, oxidative protein damage level, as well as on the expression of GFAP and EAAT1, and the secretion of cytokines from the injured astrocytes.

To the best of our knowledge, this is the first research in which the effects of the nanomaterial were assessed in an in vitro model of severe TBI. SWCNT-PEG were previously investigated in the spinal cord injury model in rats [20]. In that study, it was shown that the post-injury administration of SWCNT-PEG in the lesion site promotes axonal survival and repair, with a reduction in the lesion volume, and an increase in the number of neuronal fibers in the lesion epicenter. Additionally, a moderate recovery of motility in SWCNT-PEG-injected rats was accomplished.

### 4.1. SWCNT-PEG Do Not Affect Viability of Astrocytes Exposed to Severe Stretch Injury

With the emerging prospective applications of CNTs in biomedicine, questions regarding their potential toxicity have also been raised. Indeed, several studies have thus far exposed some toxic effects in cells following CNT exposure [30]. Firstly, it seems that the CNT toxicity is less likely to occur in cases where they were engineered and immobilized, for example on electrodes, but higher when the CNTs are utilized as free particles and not functionalized. This appears to be mainly due to the capacity of nanoparticles in general to enter the cells and disperse in the cytoplasm [31].

In our study, SWCNT-PEG did not affect the post-injury survival of astrocytes, within the first 24 h following stretch. Additionally, no overt oxidative damage to proteins extracted from the cultures astrocytes was detected, regardless of whether cells post-injury were vehicle- or SWCNT-PEG-treated.

Previously, one study observed the effects of SWCNTs on cell lines with different phagocytic properties and concluded that the higher the phagocytic activity a cell has, the higher the toxic effects will be, due to the higher uptake of the nanotubes [32]. The mechanism of toxicity is linked to the oxidative stress and formation of reactive oxygen species (ROS) [33]. It is possible that in the in vitro conditions of our research, astrocytes did not have pronounced phagocytic function, and it would be interesting to determine whether the toxicity changes in co-cultures with neurons and microglia. On the other side, in our study, we used SWCNTs functionalized with PEG that are proven to be less toxic when internalized compared to the non-functionalized SWCNTs [34]. It is important to point out that SWCNT-PEG show dose-dependent toxicity, and the concentration used in our research (5 μg/mL) has not been shown to cause a significant rise in ROS production [34].

The obtained results confirm that SWCNT-PEG are not toxic to primary astrocyte cells in culture, although they do not promote better survival of the same cells after the injury. These results also need to be critically evaluated because research methods for monitoring the increase or decrease in cell death need to be used for more than 24 h. Namely, previous studies have shown cytotoxicity of differently chemically functionalized SWCNTs after 48 h and up to 4 days. Therefore, in order to detect the true function of SWCNTs in in vitro astrocytes after injury, more cytotoxicity studies need to be conducted before switching to in vivo models.

### 4.2. Application of SWCNT-PEG to Astrocytes Exposed to Severe Stretch Injury Prevents the Decrease in the Expression of EAAT1 in Severely Stretched Astrocytes

One of the many functions of astrocytes is the regulation of extracellular glutamate via the EAAT transporters that these cells can express. This function is of key importance in brain trauma wherein large quantities of glutamate are released to induce excitotoxicity and promote cell death, and secondary brain injury [13].

The results of our study show that in vitro TBI causes a decrease in EAAT1 expression in astrocytes. This finding is in accordance with the results of previous studies, both in vitro and in vivo, which were conducted on different models of ischemic [35,36,37,38,39] and trauma-caused brain injury [14,15,40]. One of the possible reasons for this decrease could be related to changes in astrocyte morphology and subsequent downregulation of gene expression due to the cell stretching. Astrocytes are strongly adaptable cells that can quickly change their morphological and functional properties depending on their surroundings, for example in tissue damaged by TBI [41]. Several studies have confirmed that a change in the actin cytoskeleton of astrocytes is connected to the lowered expression of EAAT1 transporters [15,42,43,44].

Our results also show that the application of SWCNT-PEG to the injured astrocytes rescues stress-injury-evoked loss of EAAT1. Levels of this protein 24 h after the injury do not differ significantly from those detected in the uninjured cells, which points to the potentially protective effects of SWCNT-PEG for the brain tissue after the injury by means of the enhanced glutamate clearance by the astrocytes. So far, this effect of SWCNT-PEG has been studied only on the uninjured cells, which showed a similar effect on the EAAT1 upregulation [19]. The mechanism of this upregulation is still not clear, but a few studies have found that SWCNTs also have an effect on the cytoskeleton; more specifically, they initiate the grouping of actin into filaments and cause changes in cell surface function and the processes of endo/exocytosis [45]. It could be possible that with this remodeling, changes caused by injury, and a subsequent decrease in EAAT1 production, are mitigated or reversed.

Some of the studies found that in vivo TBI caused reactive astrogliosis, higher expression of GFAP, and a rise in EAAT1-positive astrocytes 24 h after the injury. This was followed by a prolonged decrease in signal [15,39], probably caused by the glutamate overload of astrocytes [15]. Since our results were obtained in in vitro conditions, and no changes in GFAP levels were found, they cannot be compared to these findings. However, it would be interesting to see whether the levels of EAAT1 would still be elevated in SWCNT-PEG-treated astrocytes in periods longer than 24 h. It would also be of interest to investigate if there are effects on other plasma membrane proteins to assess if the SWCNT-PEG actions shown here might be a general cell surface phenomenon.

In the previous work in vitro using SWCNT-PEG at the same concertation as used here [18,19,23], SWCNT-PEG, when applied for a prolonged time (3 or 4 days vs. 24 h here), caused an increase in GFAP and EAAT1 levels in uninjured (vs both uninjured and injured here) astrocytes grown on polyethylene-imine-coated glass strata (vs silastic membranes here). The fact that, in the present work, we did not see changes in GFAP and/or EAAT1 expression, thus, could be attributed to any individual or combined experimental differences.

### 4.3. SWCNT-PEG Modulate the Secretory Function of Astrocytes Exposed to Severe Stretch Injury

Neurons and glia, the primary cells of the CNS, communicate with each other via signals that include neurotransmitters, ions, and other extracellular molecules, and the emerging evidence points to astrocytes as secretory cells of the CNS [46]. In brain injury, astrocytes are the cells responsible for the secretion of various factors involved in signal transduction and the stimulation of the migration of other glial cells to the site of the lesion. However, in certain instances, astrocyte-secreted proteins can have detrimental effects and promote neuroinflammation and neurodegeneration, in addition to their beneficial actions that include the promotion of tissue remodeling, axonal regeneration, glial scar formation, and neural circuit rewiring [47].

Our results indicate that the stretch injury itself had no significant effect on the levels of the selected cytokines/chemokines released from astrocytes. However, in the cell culture media conditioned by injured astrocytes exposed to SWCNT-PEG, higher concentrations of axl, BLC, G-CSF, GM-CSF, IL-3-Rb, IL-4, IL-5, IL-6, IL-9, and IL-10 were detected.

Axl is a receptor tyrosine kinase that, upon binding of the growth factor GAS6, regulates numerous physiological processes such as cell survival, proliferation, migration, and differentiation. Axl can be cleaved by proteases and released as a soluble version of the protein. We detected a low expression of soluble axl in the media released from the control and injured astrocytes, but a higher expression of axl was detected in culture media of stretched astrocytes treated with SWCNT-PEG. Usually, axl is upregulated in astrocytes after TBI, and it promotes a phenotype of astrocytes that can ingest damaged neurons and other cells after TBI [48]. We found a high concentration of released axl in culture media after treatment with SWCNT-PEG, so it can be argued that this astrocytic phenotype transformation might promote the recovery of neurological cells after TBI.

IL-6 is known as a cytokine with different roles in injured cells of the CNS. It is produced at high concentrations in the injured brain. Glial-derived IL-6 is a key factor in diverse neuroinflammatory cascades, so IL-6 gene-deficient astrocytes in vitro show an altered cytokine profile compared to control cell cultures [49]. Lau and Yu [50] demonstrated that astrocytes, after scratch injury, were positively immunostained with IL-1α, IL-6, and TNFα, and the release of these cytokines into culture media was almost double that in control astrocytes. We have detected a high concentration of released IL-6 in media of injured cells treated with CNTs compared to injured and control-condition cells. This should be beneficial for recovery from injury as IL-6 is necessary for the timely resolution of injury, e.g., cerebrovascular repair after an intracerebral hemorrhage [51].

It is known that IL-4 and IL-5 can increase the amount of nerve growth factor, which is secreted by astrocytes after neural trauma, independent of cell growth [52]. IL-4 has its own role in immunity, but it has also a role in higher functions of the normal brain such as learning and memory. It has many important functions in a variety of cellular events, such as proinflammatory and anti-inflammatory effects on astrocytes depending on the treatment [53]. There is evidence that pretreatment with IL-4 in primary mouse astrocytes can decrease the production of NO and inducible NO synthase protein, as well as modulate the secretion of TNFα after liposaccharide stimulation. As we recorded an increased release of IL-4 from injured astrocytes treated with SCWNT-PEGs, this result raises an issue of whether there could be paracrine activity of this cytokine in injury or in the recovery thereof.

In our study, cytokine IL-10 was elevated in culture media of injured astrocytes treated with CNTs. This cytokine has a known immunomodulatory role after TBI. Kamm et al. [54] found that, in the brains of adult male Sprague Dawley rats, there is an elevated expression of IL-10 mRNA immediately following TBI, with levels of protein IL-10 being initially stable, but beginning to rise rapidly after 2 h. Studies with IL-10 knock-out mice showed that IL-10 is useful after TBI, but the usefulness of IL-10 administered after TBI as a therapeutic agent depends on the administration route and experimental plan [55].

It is known that the IL-9 receptor is expressed in brain cells such as microglia, astrocytes, oligodendrocyte progenitor cells, and oligodendrocytes, and its expression is induced in the brain and spinal cord during encephalomyelitis [56]. In our case, the amount of IL-9 was higher in injured astrocytes treated with CNTs. The exact role of IL-9 is a subject of ongoing investigations and remains rather unclear, as there is evidence both for its protective role in some conditions, e.g., in experimental autoimmune encephalomyelitis [57], as well as its ameliorative effects of IL-9 neutralization, e.g., in experimental strokes [58].

Results of the microarray cytokine measurement also showed a significant increase in the release of G-CSF and GM-CSF, cytokines that stimulate the production and maturation of granulocytes and, thus, support the immune system, aiding in the recovery from certain medical conditions. However, these cytokines have effects beyond simply stimulating the proliferation of neutrophils and monocytes [59]. G-CSF was found to regulate diverse functions in the nervous system and has shown ameliorative effects in certain pathological conditions, as it exhibited neurotrophic effects on primary cholinergic neurons in vitro [60], and in vivo promoted neuronal survival after focal cerebral ischemia [61,62]. One TBI retrospective study showed that plasma G-CSF levels are rapidly elevated in patients and decline after 14 days in those with mild, but not severe, brain trauma, and that higher circulating G-CSF levels correlate with better outcomes at 6 months post-injury [63]. Administration of G-CSF has also been suggested as a potential therapeutic strategy for the prevention of long-term complications of TBI, including dementia [64]. It has also been suggested that G-CSF can enhance neurological function by promoting the sprouting of the corticospinal tract into the denervated side of the cervical spinal cord. Additionally, G-CSF may mitigate microglia degeneration and restore a proper balance in synaptic pruning within the hippocampus, leading to improved neurological outcomes [65]. Similarly, studies have shown that GM-CSF may also have significant protective properties in the chronic consequences of experimental TBI [66,67].

Regarding the other cytokines that were found to be affected by the stretch injury and the nanotubes application, specifically, IL-1Rb (IL-1 receptor type II) and BLC (also known as CXCL13), limited information is available on their role in TBI pathophysiology. IL-1Rb, which comes in a membrane-bound and soluble form, is a decoy receptor, meaning that it captures IL-1 but does not initiate signal transduction, and by doing that it regulates IL-1-mediated signaling [68]. Conversely, BLC has been mostly associated with proinflammatory effects, as it was found to be frequently elevated in cerebrospinal fluid in a variety of inflammatory CNS conditions [69,70], and it has been suggested as a danger signal that is upregulated to recruit peripheral immune cells to the CNS through the damaged BBB [71]. The exact implications of our findings on the levels of excreted IL-1Rb and BLC from the injured astrocytes are difficult to define, and further research is warranted.

Taken together, our results indicate that the SWCNT-PEG effect on cytokines released from injured astrocytes is aligned with promoting recovery after injury. Should SWCNT-PEG be used in the treatment of the TBI, this nanomaterial would not only encounter injured but also uninjured astrocytes. Consequently, we tested the effect that SWCNT-PEG may have on cytokine release from uninjured astrocytes. We found the change in the level of the sole cytokine, i.e., the decrease in the release of MIP-1 gamma (also referred to as CCL9, i.e., chemokine ligand 9, macrophage inflammatory protein-related protein-2 and CCF 18) from SWCNT-PEG-treated uninjured astrocytes. As this protein plays a role in the development of the TBI in a mouse model [72], its decrease in the surrounding uninjured astrocyte may point out to a potential and additional protective action of SWCNT-PEG in the parenchyma surrounding the TBI site. While out of scope of the present study, of additional interest would be to compare the cytokine release pattern from astrocytes exposed to SWCNT-PEG to that of astrocytes exposed to known astrocytic secretagogues, such as adenosine triphosphate. There is a three-pronged rationale for this endeavor: (i) this nucleotide is increased in the extracellular space in experimental TBI [73]; (ii) it can cause glutamate release from cultured uninjured astrocytes via regulated exocytosis, i.e., vesicular release [74,75]; and SCWNT-PEGs affect vesicular recycling in cultured uninjured astrocytes [76].

## 5. Conclusions

We studied the effects of SWCNT-PEG administration on cultured astrocytes that were subjected to in vitro stretch injury. Our results showed that SWCNT-PEG do not increase cell death or oxidative damage to proteins in injured cells. We also showed that SWCNT-PEG prevented the loss of the plasmalemmal transporter EAAT1 in injured cells, which could point to the enhanced protective role of astrocytes against glutamate toxicity in brain injury. Additionally, SWCN-PEGs altered the cytokine secretory function of astrocytes aligned with promoting recovery after injury, which points to the possible mechanism of their protective effects in CNS injury by affecting the brain tissue microenvironment. Future additional studies regarding the possible long-term toxicity of SWCNTs on different cell types of neuronal origin are necessary, as well as tests to determine if the results from in vitro investigations translate to the in vivo model of TBI.

## Figures and Tables

**Table 1:** Information on used antibodies.

Antibody	Immunogen	Manufacturer; Catalog Number; RRID; Host Species and Antibody Type	Dilution
anti-GFAP	native GFAP purified from pig spinal cord	Cell Signaling Technology (Danvers, MA, USA); 3670; AB_561049; mouse monoclonal	1:1000
anti-EAAT1	synthetic peptide corresponding to residues surrounding Glu230 of human EAAT1	Cell Signaling Technology; 5685; AB_10694915; rabbit monoclonal	1:1000
anti-β-actin	β-actin (C4) raised against gizzard actin from chicken	Santa Cruz Biotechnology (Dallas, TX, USA); sc-47778; AB_2714189; mouse monoclonal	1:1000
biotinylated goat anti-rabbit	gamma immunoglobulins	Thermo Fisher Scientific; 65-6140; AB_2533969; goat polyclonal	1:3000
biotinylated goat anti-mouse	gamma immunoglobulins	Thermo Fisher Scientific; A16070; AB_2534743; goat polyclonal	1:3000

Abbreviations: RRID, research resource identifier.

## Data Availability

The data that support the findings of this study are available within the article and Supplementary Materials or upon request from the corresponding author.

## Supplementary Materials

The following supporting information can be downloaded at: https://www.mdpi.com/article/10.3390/cells13030225/s1, Figure S1: Purity of primary astrocyte culture; Scheme S1: Schematic illustration of the SWCNT-PEG structure; Figure S2: AFM height (left) and amplitude (right) images of SWCNT-PEG; Figure S3: Mid-infrared spectra of SWCNT-PEG and the starting carbon nanotube materials for the functionalization SWCNT-COOH; Figure S4: Thermogravimetric curve showing weight loss of SWCNT-PEG as a function of temperature during heating at 5 °C/min in air; Figure S5: Ultraviolet-visible–near-infrared red spectra of SWCNT-PEG and the starting carbon nanotube materials for the functionalization of SWCNT-COOHs; Table S1: Results of the multiplex cytokine (listed in alphabetical order) array analysis of culture media samples from control (uninjured and PEG-treated) or injured astrocytes, the latter treated with the vehicle PEG (sTBI) or SWCNT-PEG (sTBI+CNTs); Figure S6: The effects of chemically functionalized single-walled carbon nanotubes (CNTs) on the protein expression of (A) GFAP and (B) EAAT1 in mouse primary astrocytes; Figure S7: The effects of SWCNT-PEG on the secretion of selected cytokines from primary mouse astrocytes; Table S2: Results of the multiplex cytokine (listed in alphabetical order) array analysis of culture media samples from control, non-treated astrocytes, and cells treated with vehicle (PEG) or SWCNT-PEG (CNTs).
