# Supplementary material for: Chemically Functionalized Single-Walled Carbon Nanotubes Prevent the Reduction in Plasmalemmal Glutamate Transporter EAAT1 Expression in, and Increase the Release of Selected Cytokines from, Stretch-Injured Astrocytes in Vitro
Source: Cells. 2024 Jan 25;13(3):225. doi: 10.3390/cells13030225 (PMC10854924; doi:10.3390/cells13030225)
Supplement: Supplementary file 1 [file cells-13-00225-s001.zip › cells-2490923-supplementary.pdf]

Chemically functionalized single-walled carbon nanotubes prevent the reduction in plasmalemmal glutamate transporter EAAT1 expression in, and increase the release of selected cytokines from, stretch-injured astrocytes *in vitro*

Nika Gržeta Krpan, Anja Harej Hrkać, Tamara Janković, Petra Dolenec, Elena Bekyarova, Vladimir Parpura, and Kristina Pilipović

Supplementary information:

1. **Immunocytochemistry on glial fibrillary acidic protein (GFAP) and excitatory amino acid transporter 1 (EAAT1) to assess cell culture purity.**

For the immunocytochemistry analyses, purified astrocytes were detached from flasks and seeded onto silastic membranes. Astrocytes attached to the membranes were rinsed twice with 0.01 M phosphate buffered saline (PBS) and fixed, using 4 % paraformaldehyde in PBS for 20 min. For the permeabilization, 0.2% Triton-X in PBS (PBS-TX) was used for 20 min on astrocytes to be immunostained for GFAP, while this step and the addition of detergent (Triton-X) in subsequent steps of the protocol were omitted for the EAAT1 staining. Astrocytes were then incubated with 3 % BSA in PBS or PBS-TX for 1 h to block non-specific antibody binding. Incubation with primary mouse anti-GFAP (Table 1; 1:1000 dilution) or rabbit anti-EAAT1 (Table 1; but at the dilution of 1:200) was done overnight at 4 °C. Afterwards, astrocytes were washed in PBS and incubated for 2 h with the secondary Alexa Fluor™ 488-conjugated goat anti-mouse (Cell Signaling Technology, Danvers, MA, USA; Cat. No. 4408; RRID: AB_10694704) or DyLight™ 594-conjugated goat anti-rabbit (Abcam, Cambridge, MA, USA; Cat. No. ab96901; RRID:AB_10679699) polyclonal antibodies, both at 1:200 dilution in PBS-TX or PBS, respectively. Membranes containing astrocytes were rinsed and mounted onto glass slides using the Mowiol mounting medium (Merck Millipore, Billerica, MA, USA). Astrocytes were visualized using an Olympus IX73 Inverted Microscope equipped with a LUCPlan FLN 40X/0.60 Ph2 objective (Olympus Corporation, Tokyo, Japan). Microphotographs were captured by the CellSense Imaging Software (Olympus Corporation, Tokyo, Japan) and analyzed by ImageJ software (NIH,

Bethesda, MD, USA). The cultures contained >99% of astrocytes based on GFAP and EAAT1 immunostaining (Figure S1).

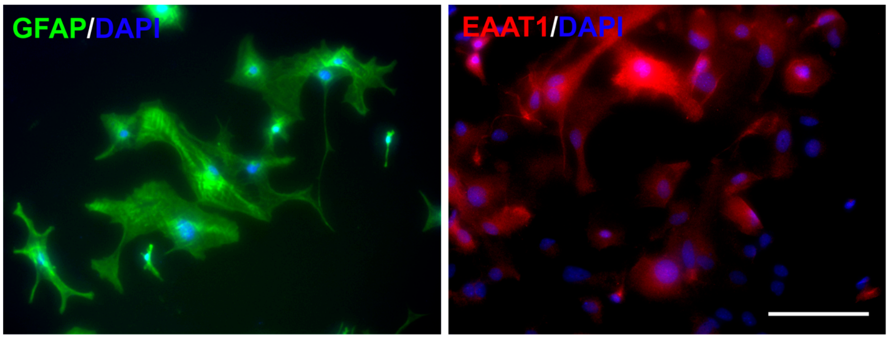

Figure S1. Purity of primary astrocyte culture. Immunolabeling of primary mouse astrocyte cultures with the markers GFAP (green) and EAAT1 (red) revealed pure primary astrocyte culture. Nuclei are stained with 4',6'-diamidino-2-phenylindole (DAPI) (blue). Scale bar: 100 μm.

2. Polyethylene glycol functionalized single-walled carbon nanotubes

Single-walled carbon nanotubes (SWCNTs) were functionalized with the polyethylene glycol (PEG) chain of 600 $gmol^{-1}$ molecular mass. The covalent attachment of the PEG chain was achieved by reacting the carboxylic groups of the SWCNTs (introduced by reaction of the SWCNTs with nitric acid) with the hydroxyl group of PEG. Scheme S1 shows a simplified schematic illustration of the SWCNT-PEG structure.

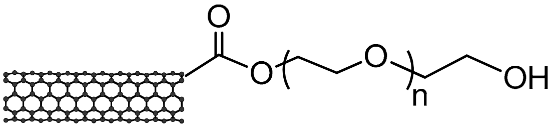

Scheme S1. Schematic illustration of the SWCNT-PEG structure.

The procedure for PEG functionalization of SWCNTs is described below.

Purified electric-arc-produced single-wall carbon nanotubes (SWCNTs) with carboxylic acid functionality (SWNT-COOH, P3-SWNT) were obtained from Carbon Solutions, Inc. (www.carbonsolution.com) (Riverside, CA, USA). P3-SWNT material (1 g) was dispersed in dry

dimethylformamide (DMF; 1 L) by ultrasonication for 2 h and high-shear mixing for 30 min to give a homogeneous suspension. The SWCNT solute was transferred to a round-bottom flask and purged with argon. Oxalyl chloride (20 mL) was added dropwise to the SWCNT solute at 0°C under argon to convert the carboxylic acid groups to acyl chloride. The reaction mixture was stirred for 1 hour at 0 °C and 2 hours at room temperature and then heated to 70 °C for 12 hours to remove excess oxalyl chloride (boiling point 63 °C). The functionalization was performed by adding PEG (10 g) at room temperature and the mixture was heated at 120°C for 5 days. During this step the acyl chloride groups on the SWCNTs react with the OH-groups of the PEG resulting in covalent attachment of the polymer via an ester bond. After cooling to room temperature, the mixture was filtered through a 0.22-μm Teflon membrane, washed with DMF and distilled water. SWCNT-PEG contained 72.3 weight % (wt%) SWCNTs, 22.6 wt% PEG and 5.1 wt% metal.

Due to the presence of PEG, the functionalized nanotube material can be dispersed in water by utrasonication. Stock solution of SWCNT-PEG aqueous colloidal solute (2.0 mg/mL) was prepared by dispersing dry powder of the functionalized carbon nanotubes in purified water under ultrasonication (bath sonicator Aquasonic 50HT, VWR Scientific, Radnor, PA, USA; sonic power 75 W, frequency ~ 40 kHz). Further dilution of this stock solute was done either in purified water (for below characterization of this material) or in cell culture media (for use in experiments with astrocytes).

The material was characterized using Atomic Force Microscopy (Figure S2) to determine the nanotube morphology, mid-infrared spectroscopy (Figure S3) to assess the functional groups, thermogravimetric analysis (Figure S4) to estimate the content of remaining metal, and ultraviolet-visible-near infrared spectroscopy (Figure S5) to verify that the SWCNTs retained structural and electronic integrity after the functionalization.

Atomic Force Microscopy (AFM) was performed with a Digital Instruments Nanoscope IIIA (Digital Instruments, Santa Barbara, CA, USA) in a tapping mode using an n^+-silicon cantilever with a spring constant of 40 Nm^{-1}. The samples for AFM observation were prepared on a mica

stratum by adding a drop of a diluted SWCNT-PEG aqueous solute and allowing it to dry in air. SWCNT-PEG exist as individual nanotubes or their small bundles (Figure S2). The individual nanotubes have a diameter of 1.5 nm, while small bundles (axially aligned group of nanotubes) have a diameter of 2-7 nm; the length of SWCNT-PEG is in the range of 0.3 – 1.5 μm.

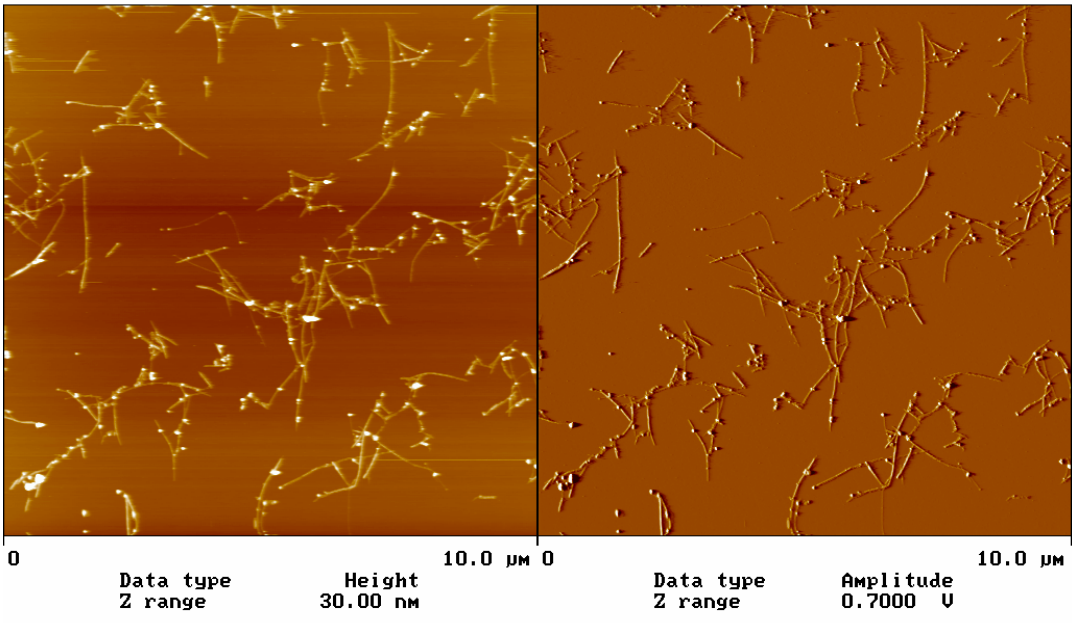

Figure S2. AFM height (left) and amplitude (right) images of SWCNT-PEG.

Mid infrared spectroscopy was used to identify the chemical groups present in SWCNT-PEG. Fourier transform infrared (FT-IR) spectra were obtained using a Nicolet Nexus 670 FT-IR spectrometer (Thermo-Nicolet, Madison, WI, USA) at 8 cm^{-1} resolution and 200 scans in the spectral range of 400-4000 cm^{-1}. Carbon nanotube aqueous solutes were deposited on a silicon stratum and dried in air at 90 °C. Figure S3 compares the spectra of SWCNT-PEG and the starting material for the functionalization, carboxylic acid group functionalized SWCNTs (SWCNT-COOH). The spectrum of SWCNT-COOH shows a signal at 1723 cm^{-1}, which is due to the carbonyl stretch of the carboxylic acid groups and a broad band from 3100 cm^{-1} to 3600 cm^{-1} assigned to -OH groups in -COOH. In the spectrum of SWCNT-PEG the carbonyl (C=O) stretch is shifted to 1740 cm^{-1}, which is representative of the ester bond formed between the carboxylic acid groups of the nanotubes and OH-groups of PEG confirming the covalent attachment of PEG to SWCNTs. The spectrum of SWCNT-PEG also exhibits the characteristic stretching vibration of

C-O-C of an ester bond, a broad band due to the O-H vibration in PEG around 3470 cm^{-1} and a C=C stretch of double bonds located near the introduced oxygen-containing groups in SWCNTs at 1590 cm^{-1}.

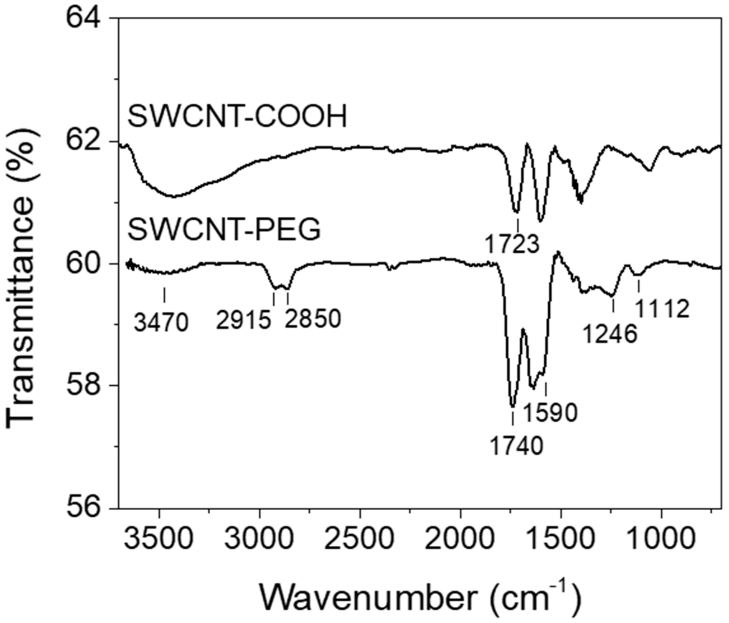

Figure S3. Mid-infrared spectra of SWCNT-PEG and the starting carbon nanotube materials for the functionalization SWCNT-COOH.

The thermogravimetric analysis (TGA) of SWCNT-PEG was recorded on a Pyris 1 Thermogravimetric Analyzer (Perkin-Elmer, Norwalk, CT, USA) in air at a heating rate of 5 °C/min. The weight loss as a function of temperature is shown in Figure S4. The SWCNTs are produced by the electric arc method using a catalyst containing nickel (Ni) and yttrium (Y) in a mass ratio of 2.6 to 1 (atomic ratio 4:1). Small amount of the metals remains after the SWCNT purification, which is detected in the TGA as a residue after burning the nanotubes in air at high temperature. From the residue at 900°C it is estimated that the SWCNT-PEG material contains 5.1 wt% metal (Me), *i.e.*, 3.6% Ni and 1.5 weight % Y.

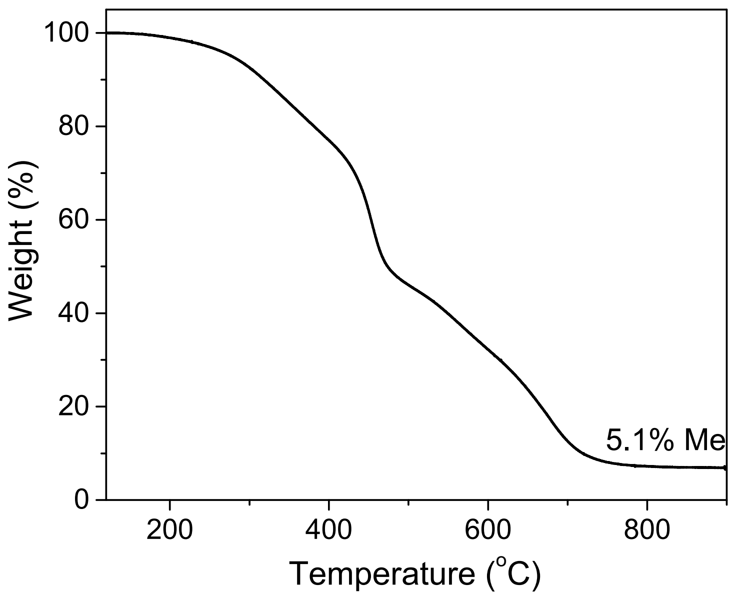

Figure S4. Thermogravimetric curve showing weight loss of SWCNT-PEG as a function of temperature during heating at 5 °C/min in air.

The ultraviolet-visible-near infrared (UV-Vis-NIR) spectrum of a diluted SWCNT-PEG aqueous solute was recorded on a Varian CARY 500 UV-Vis-NIR spectrophotometer (Varian Inc., Palo Alto, CA, USA) in the spectral range of 7000 - 35000 cm^{-1} (Figure S5). The spectrum of SWCNT-PEG shows the characteristic interband transitions of single-wall carbon nanotubes in the NIR region – the second semiconducting (S_{22}) and the first metallic (M_{11}) transitions. The same characteristic bands are observed in the spectrum of SWCNT-COOH, which were used as the starting SWCNT material for the functionalization. The intensity of these peaks is related to the content of carbon nanotubes in the solute. In combination with a determination of metal content by TGA, this allows a full analysis of the composition of the SWCNT-PEG samples.

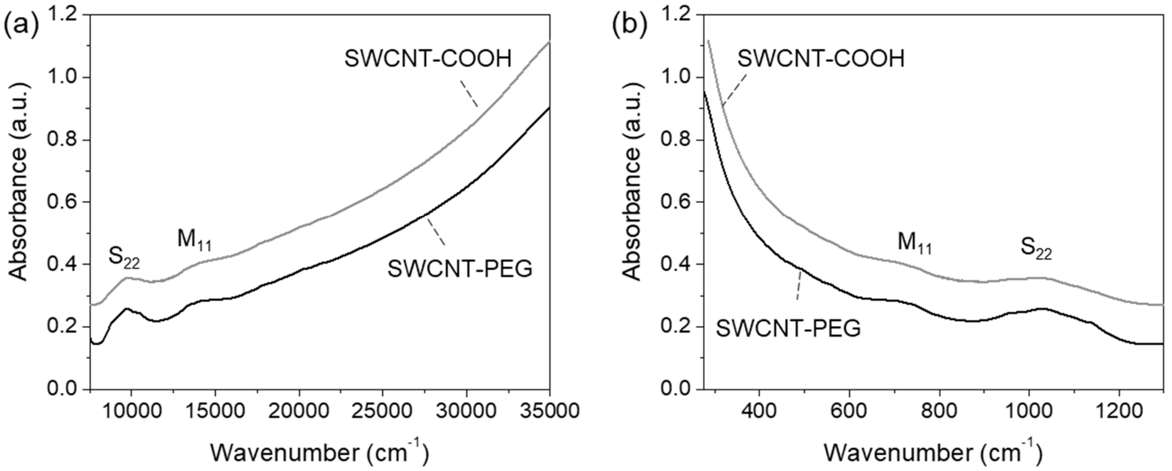

Figure S5. Ultraviolet-visible-near infrared red spectra of SWCNT-PEG and the starting carbon nanotube materials for the functionalization SWCNT-COOH.

3. Results of the cytokine analyses

Table S1. Results of the multiplex cytokine (listed in alphabetical order) array analysis of culture media samples from control (uninjured and PEG treated) or injured astrocytes, the latter treated with the vehicle PEG (sTBI) or SWCNT-PEG (sTBI+CNTs). Reported *p*-values are for one-way ANOVA, F (2,6).

	**Control**			**sTBI**			**sTBI+CNTs**			
	Mean	±	SEM	Mean	±	SEM	Mean	±	SEM	***p*-value**
axl	16718	±	3275	16744	±	992	32863	±	4688	*0.022*
BLC	72646	±	13101	68306	±	7685	114230	±	11023	*0.044*
CD30L	40834	±	7406	37347	±	2129	57518	±	8129	0.140
CD30/TNFRSF8	17634	±	3056	14769	±	550	32816	±	7926	0.082
CD40	12789	±	3739	13767	±	2140	27983	±	14188	0.426
CRG-2	70256	±	16899	61489	±	3534	132431	±	40265	0.178
CTACK	168821	±	25588	149857	±	27855	209095	±	13274	0.259

CXCL16	135924	±	31167	121224	±	12164	206239	±	22130	0.084
Eotaxin	17274	±	3933	16108	±	2149	25180	±	6360	0.363
Eotaxin-2	78016	±	14912	76439	±	3788	152648	±	40262	0.121
FAS ligand	13528	±	1343	9451	±	1178	22810	±	5006	0.053
Fractalkine	218289	±	46065	182008	±	17973	345859	±	46507	0.056
G-CSF	7481	±	1267	4680	±	184	14255	±	3482	*0.048*
GM-CSF	51936	±	11738	45898	±	7099	89457	±	10259	*0.041*
IFN-gamma	17532	±	3378	16572	±	2432	21392	±	3084	0.521
IGF-BP-3	448402	±	88861	390930	±	17913	604411	±	108635	0.240
IGF-BP-5	133681	±	34500	115008	±	16867	175032	±	18254	0.284
IGF-BP-6	80456	±	20720	69400	±	9763	169463	±	56161	0.166
IL1-alpha	112183	±	28635	91247	±	14727	215497	±	62762	0.148
IL1-beta	8186	±	2231	8152	±	1474	19227	±	4960	0.084
IL2	38458	±	6567	32970	±	6227	58370	±	7362	0.081
IL3	11386	±	2404	8363	±	929	11350	±	1123	0.380
IL3 Rb	19144	±	2364	24779	±	1053	32123	±	3800	*0.037*
IL4	214442	±	28355	180581	±	6772	346468	±	43122	*0.018*
IL5	16926	±	3241	14009	±	1939	30137	±	3592	*0.019*
IL6	25709	±	5055	20698	±	2874	42066	±	2905	*0.016*
IL9	65888	±	14082	51442	±	9205	101808	±	7296	*0.037*
IL10	11673	±	1375	9087	±	1193	17373	±	975	*0.007*
IL12-p40/p70	19702	±	4236	18016	±	3715	27584	±	1589	0.183
IL12-p70	110394	±	12739	104720	±	27665	214345	±	58593	0.152
IL13	59626	±	13223	50348	±	6015	97722	±	19530	0.114
IL17	9609	±	2794	8648	±	795	21571	±	7935	0.195
KC	109413	±	20505	100752	±	12739	213106	±	54250	0.107
Leptin R	35408	±	8290	33626	±	3933	72109	±	22581	0.173
Leptin	9897	±	740	7366	±	914	11966	±	2075	0.139

LIX	113842	±	12851	112966	±	13461	186224	±	28028	0.060
L-Selectin	14702	±	2472	13238	±	2333	24567	±	4951	0.116
Lymphotactin	83926	±	19325	63803	±	5413	120520	±	18534	0.107
MCP-1	239798	±	49964	205393	±	40509	359565	±	8713	0.060
MCP-5	101423	±	20104	72998	±	9999	139967	±	12897	0.052
M-CSF	210871	±	45931	137746	±	25395	268783	±	19732	0.075
MIG	15348	±	2471	9842	±	2483	18248	±	1373	0.084
MIP-1-alpha	47635	±	10362	35379	±	5943	68237	±	6437	0.064
MIP-1-gamma	307346	±	69618	205631	±	21454	327544	±	43082	0.246
MIP-2	214940	±	37607	173253	±	23345	339213	±	49901	0.052
MIP-3-beta	61684	±	10553	54333	±	6345	110907	±	21456	0.061
MIP-3-alpha	15641	±	2084	9752	±	1472	19260	±	4647	0.166
PF4	168461	±	32929	121861	±	17546	225070	±	51474	0.218
P-Selectin	120004	±	20395	96472	±	9785	151060	±	29478	0.272
RANTES	38955	±	6024	29829	±	3325	43090	±	8244	0.365
SCF	23807	±	4102	18179	±	2934	25900	±	6101	0.507
SDF-1-alpha	113183	±	28619	87605	±	19485	163462	±	28163	0.187
TARC	115059	±	30467	80720	±	14699	153396	±	21837	0.167
TCA-3	247875	±	52345	170076	±	21707	296098	±	35259	0.144
TECK	19522	±	2821	14837	±	1203	22342	±	1732	0.099
TIMP-1	126358	±	20060	112581	±	18140	155062	±	10080	0.262
TNF-alpha	20712	±	4749	20915	±	3476	28785	±	2109	0.273
sTNF RI	147838	±	28012	109439	±	11065	193438	±	20422	0.079
sTNF RII	121439	±	16069	90405	±	5325	147920	±	20473	0.097
TPO	63183	±	9848	57808	±	7192	84677	±	14297	0.257
VCAM-1	166884	±	35232	127283	±	8685	213899	±	41508	0.236
VEGF	15228	±	3170	13308	±	1365	20346	±	5154	0.412

Abbreviations: axl, tyrosine kinase receptor Axl; BLC, B lymphocyte chemoattractant; CD30L, CD30 ligand; CD30/TNFRSF8, tumor necrosis factor ligand superfamily member 8; CD40, cluster of differentiation 40; CRG-2, cytokine-responsive gene-2; CTACK, cutaneous T cell-attracting chemokine; CXCL16, chemokine (C-X-C motif) ligand 16; G-CSF, granulocyte colony-stimulating factor; GM-CSF, granulocyte-macrophage colony-stimulating factor; IFN-gamma, interferon gamma; IGF-BP-3, insulin-like growth factor-binding protein 3; IGF-BP-5, insulin-like growth factor-binding protein 5; IGF-BP-6, insulin-like growth factor-binding protein 6; IL1-alpha, interleukin 1 alpha; IL1-beta, interleukin 1 beta; IL2, interleukin 2; IL3, interleukin 3; IL3 Rb, interleukin 3 receptor beta; IL4, interleukin 4; IL5, interleukin 5; IL6, interleukin 6; IL9, interleukin 9; IL10, interleukin 10; IL12, interleukin 12; IL13, interleukin 13; IL17, interleukin 17; KC, keratinocyte-derived cytokine; LIX, lipopolysaccharide-induced CXC chemokine; MCP-1, monocyte chemoattractant protein-1; MCP-5, monocyte chemoattractant protein-5; M-CSF, macrophage colony-stimulating factor; MIG, monokine induced by gamma; MIP-1-alpha, macrophage inflammatory protein-1 alpha; MIP-1-gamma, macrophage inflammatory protein-1 gamma; MIP-2, macrophage inflammatory protein-2; MIP-3-beta, macrophage inflammatory protein-3 beta; MIP-3-alpha, macrophage inflammatory protein-3 alpha; PF4, platelet factor 4; RANTES, regulated on activation, normal T cell expressed and secreted; SCF, stem cell factor; SDF-1-alpha, stromal cell-derived factor 1 alpha; TARC, thymus- and activation-regulated chemokine; TCA-3, T-cell activation protein 3; TECK, thymus-expressed chemokine; TIMP-1, tissue inhibitor of metalloproteinases-1; TNF-alpha, tumor necrosis factor alpha; sTNF RI, human soluble tumor necrosis factor receptor I; sTNF RII, human soluble tumor necrosis factor receptor II; TPO, thrombopoietin; VCAM-1, vascular cell adhesion molecule-1; VEGF, vascular endothelial growth factor.

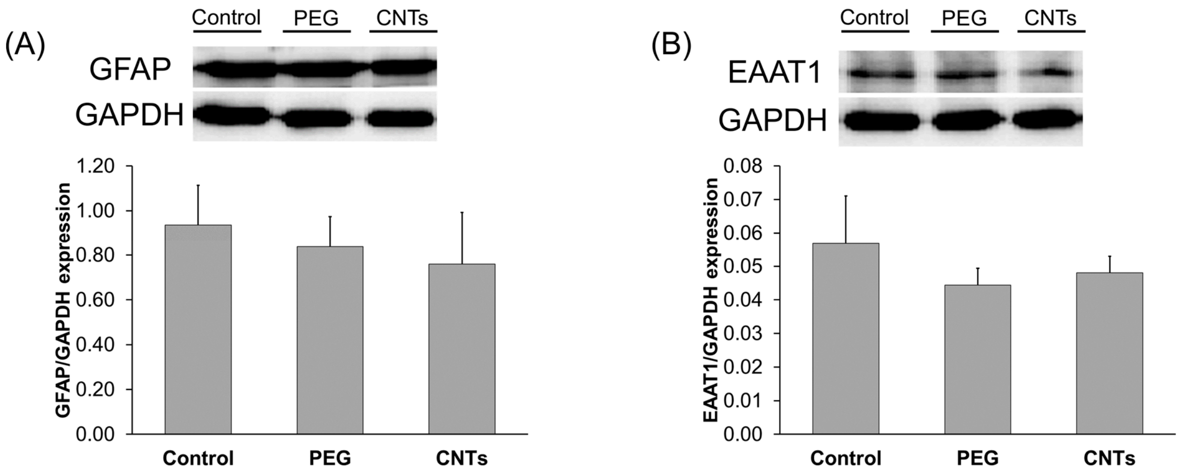

Figure S6. The effects of chemically functionalized single-walled carbon nanotubes (CNTs) on the protein expression of (A) GFAP and (B) EAAT1 in mouse primary astrocytes. Cells were either untreated (Control) or exposed to the vehicle (PEG at 1 μg/ml. i.e., at the concentration corresponding to 20 wt% of the SWCNT-PEG solute) or CNTs (5 μg/ml of SWCNT-PEG) for 23 h, equivalent to the duration of the treatment of the stretch-injured cells, with the aim to determine the effects of the vehicle and the investigated nanomaterial on astrocytes not exposed to the stretch paradigm. There were no changes in the expressions of GFAP or EAAT1 proteins between astrocytes treated with CNTs or PEG compared to the control, untreated cells. Results of the densitometric analyses were corrected for the GAPDH contents. Data are expressed as mean ± SEM (N = 3-4).

(A) (B)

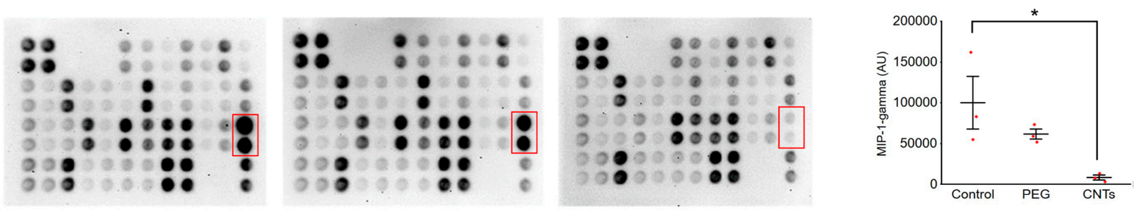

(C)

	A	B	C	D	E	F	G	H	I	J	K	L
1<br>2	POS	POS	NEG	NEG	Blank	BLC	CD30L	Eotaxin	Eotaxin-2	FAS ligand	Fractalkine	G-CSF
3<br>4	GM-CSF	IFN-gamma	IL1-alpha	IL1-beta	IL2	IL3	IL4	IL6	IL9	IL10	IL12-p40/p70	IL12-p70
5<br>6	IL13	IL17	I-TAC	KC	Leptin	LIX	Lymphotactin	MCP-1	M-CSF	MIG	MIP-1-alpha	MIP-1-gamma
7<br>8	RANTES	SDF-1	TCA-3	TECK	TIMP-1	TIMP-2	TNF-alpha	sTNF RI	sTNF RII	Blank	Blank	POS

Figure S7. The effects of SWCNT-PEG on the secretion of selected cytokines from primary mouse astrocytes. (**A**) Representative membranes showing the content/pattern of cytokines, released from the untreated group of astrocytes (Control), PEG-treated cells (PEG), and astrocytes subjected to SWCNT-PEG treatment (CNTs). (**B**) Summary graphs showing significant decrease in signal intensity of the only cytokine, MIP-1 gamma (boxed in A and C), in the cell culture media samples from the SWCNT-PEG treated astrocytes. Data are expressed as mean ± SEM. *P < 0.05. (**C**) Table with the list of cytokines available within the Mouse Inflammation Array C1 kit simultaneously assessing 40 mouse cytokines. Letters (A-L) and numbers (1-8) indicate coordinates (x,y) on the dot-blot. Colored boxes indicate the only protein with significant change in its expression.

Table S2. Results of the multiplex cytokine (listed in alphabetical order) array analysis of culture media samples from control, non-treated astrocytes, and cells treated with vehicle (PEG) or SWCNT-PEG (CNTs). Reported *p*-values are for one-way ANOVA, F (2,6).

	**Control**			**PEG**			**CNTs**			
	Mean	±	SEM	Mean	±	SEM	Mean	±	SEM	***p*-value**
BLC	12560	±	3015	12481	±	1809	10658	±	1367	0.791
CD30L	6816	±	1474	5824	±	886	5772	±	598	0.742
Eotaxin	2670	±	717	1910	±	145	1701	±	302	0.353
Eotaxin-2	8900	±	1374	8638	±	604	8864	±	766	0.979
FAS ligand	2846	±	189	2742	±	110	2687	±	278	0.858
Fractalkine	15659	±	1178	15866	±	1025	17410	±	271	0.394
G-CSF	2504	±	62	3050	±	429	2983	±	431	0.528
GM-CSF	8466	±	2400	7473	±	1065	6487	±	925	0.700
IFN-gamma	2330	±	405	1898	±	226	1136	±	138	0.059
IL1-alpha	23515	±	4326	21113	±	1366	18503	±	1893	0.500
IL1-beta	1862	±	205	2371	±	930	1060	±	303	0.336
IL2	4488	±	1028	4381	±	151	3934	±	640	0.844
IL3	1913	±	501	2295	±	152	1956	±	314	0.716
IL4	31027	±	11283	18523	±	3996	11987	±	2978	0.237
IL6	2819	±	428	2612	±	334	1986	±	246	0.280
IL9	7597	±	799	7781	±	184	8436	±	621	0.603
IL10	1744	±	121	2132	±	171	1863	±	207	0.324
IL12-p40/p70	3070	±	544	3006	±	626	3283	±	791	0.954
IL12-p70	16631	±	983	15564	±	1239	17786	±	1237	0.449
IL13	6324	±	1505	6905	±	958	7039	±	998	0.903
IL17	2336	±	355	2610	±	220	2006	±	368	0.460
I-TAC	2797	±	471	2618	±	166	2058	±	235	0.303

KC	16338	±	3828	16306	±	1882	13862	±	3390	0.821
Leptin	1306	±	347	1339	±	146	1258	±	297	0.979
LIX	42406	±	7081	38489	±	3903	33905	±	2487	0.508
Lymphotactin	11457	±	1088	11111	±	752	10266	±	782	0.642
MCP-1	22598	±	1744	20876	±	3223	24203	±	3872	0.757
M-CSF	26969	±	2142	30575	±	716	28239	±	2334	0.438
MIG	1023	±	242	1521	±	253	1533	±	252	0.325
MIP-1-alpha	9421	±	2369	7722	±	753	10407	±	2559	0.666
MIP-1-gamma	100010	±	32148	61422	±	6244	8175	±	2957	*0.038*
RANTES	10348	±	1620	8082	±	212	5814	±	1087	0.079
SDF-1	7165	±	1010	5958	±	291	5038	±	477	0.157
TCA-3	32171	±	5981	29170	±	2886	29705	±	2865	0.866
TECK	2424	±	187	4090	±	655	4439	±	724	0.098
TIMP-1	5416	±	517	6486	±	614	4841	±	51	0.112
TIMP-2	5168	±	391	5111	±	411	4079	±	183	0.115
TNF-alpha	4538	±	353	5264	±	457	4890	±	118	0.381
sTNF RI	40916	±	4356	36272	±	1380	40744	±	3058	0.538
sTNF RII	20143	±	3752	20787	±	1113	29477	±	4157	0.162

Abbreviations. As in Table S1 with the exception of I-TAC, interferon-inducible T cell alpha chemoattractant.
